# Effects of a Physical Education Intervention on Academic Performance: A Cluster Randomised Controlled Trial

**DOI:** 10.3390/ijerph17124287

**Published:** 2020-06-16

**Authors:** Rodrigo Antunes Lima, Fernanda Cunha Soares, Jorge Bezerra, Mauro Virgílio Gomes de Barros

**Affiliations:** 1 Institute of Sport Science, University of Graz, Mozartgasse 14, 8010 Graz, Austria; 2 Research Group on Lifestyles and Health, University of Pernambuco, Arnóbio Marquês Street, 310, Recife 50100-130, PE, Brazil; fercsoares@gmail.com (F.C.S.); jorge.bezerra@upe.br (J.B.); mauro.barros@upe.br (M.V.G.d.B.)

**Keywords:** adolescent, physical activity, intervention study

## Abstract

Background: We investigated the effects of three different interventions on academic performance in students enrolled in the first year of high school. Methods: This was a cluster randomised controlled trial conducted with 1200 students enrolled in the first year of high school. Schools were randomly assigned to: 1. Doubling physical education (PE) classes (3:20 h of PE/week); 2. workshop with the PE teachers; 3. workshop with the PE teachers and doubling the PE classes; and 4. control group (1:40 h of PE/week). We assured that the schools within the groups were equal regarding: The structural condition of the sports court; number of PE teachers; number of school classes; and the average number of students per classroom. Results: Overall, the intervention was not effective in improving the students’ academic performance. However, the subgroup analysis showed that the workshop intervention group increased the academic performance of students who had failed an academic year (from 16 years of age), compared to their peers in the doubling the PE classes (1.3 points on average) and the control groups (1.4 points on average). Conclusions: Enhancing the pedagogical skills of the teachers is a promising approach in improving the academic performance of students who failed an academic year.

## 1. Introduction

Although schools are identified as an ideal setting for stimulating children and adolescents’ development and health [1], there are a limited number of randomised controlled trials evaluating the impact of school-based physical activity interventions on the students’ academic performance [2]. Since observational cohort studies and small controlled laboratory studies showed some promising positive associations between higher physical activity, motor competence and fitness, and better academic performance [2,3,4,5,6,7,8,9], it is plausible that school-based interventions via the physical education lessons impact the students’ academic performance. However, large school-based intervention studies reported conflicting results [10,11,12,13,14,15].

Most school-based interventions focused on improving the academic performance of the students by increasing the amount and/or enhancing the quality of the physical education (PE) classes [10,11,12,13,14,15], whereas other investigations implemented multicomponent interventions [16,17,18,19]. Independent of the intervention approach, results were inconsistent. Null, positive, and negative effects on academic performance were reported [2,3,4,13]. It was not clear which approach was more appropriate at influencing the students’ academic performance. In addition, some studies only observed effects on academic performance in a subgroup of students [15,17,19]. For example, Resaland et al. (2016) reported higher academic performance after a physical activity intervention only in students with the poorest academic performance at baseline [17]. Interestingly, the 2019 Nobel Prize of economics valued the contributions of three scientists who conducted a series of studies targeting poverty [20,21]. Among several means of diminishing poverty, focusing on the students in the worst situation was one of the most effective approaches in improving the students’ academic performance [20,21,22,23]. Therefore, besides evaluating the effects of school-based interventions on the students’ academic performance, it was necessary to evaluate whether students with problems at school (i.e., older students who have failed an academic year) were benefiting from the interventions. Thus, we investigated the effects of three different interventions on the academic performance of students enrolled in the first year of high school. In addition, we also evaluated the effects of the interventions on the academic performance of students based on their age at the beginning of the academic year, as an indicator that the student has missed an academic year. 

## 2. Materials and Methods

This was a cluster randomised controlled trial, part of the SACODE Project, which was an intervention in physical education classes to reduce sedentary behaviour and improve cognitive function. A total of eleven high schools were in the vale do Capibaribe region in the Pernambuco state in Brazil, and all were eligible for participating in the study. The protocol was approved by the Human Research Ethics Committee of the University of Pernambuco (protocol no. 55741016.0.0000.5207) and registered in the Brazilian Clinical trial registry: RBR-88tgky. 

### 2.1. Randomisation

**Figure 1:**
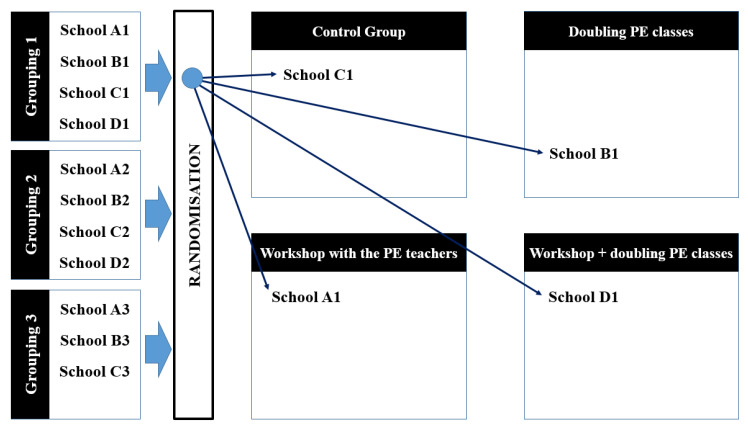
Randomisation of the schools.

A researcher randomly assigned schools ensuring that groups would be equal in regards to: (1) The structural condition of the sports court (covered or uncovered); (2) number of PE teachers; (3) number of school classes; and (4) the average number of students per classroom. This categorisation resulted in three homogenous groupings of schools based on the aforementioned criteria, which is illustrated in Figure 1. After grouping the schools, we randomly assigned schools within each grouping to the following groups: Control group, doubling PE classes; workshop with the PE teachers; and workshop with PE teachers + doubling PE classes (Figure 1).

### 2.2. Participants

**Figure 2:**
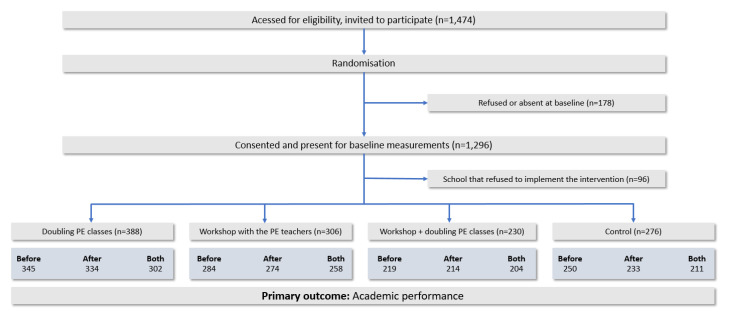
Flow of individual participants through the study with primary outcome measures.

All the 1474 students enrolled in the first year of high school were invited to participate. At the baseline, 1296 students (572 boys and 724 girls) who have returned the informed consent signed by their parents/guardians were included in the SACODE Project. However, after baseline measures, one school that should have doubled the amount of PE classes did not agree to implement the intervention and was excluded from this study. Therefore, 96 students who were enrolled in this school were excluded. Figure 2 presents a flowchart containing information on the number of adolescents with complete data in each step of the study.

### 2.3. Intervention

**Figure 3:**
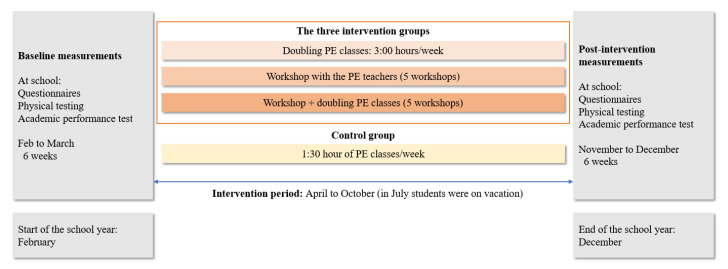
Content and timetable of intervention and measurements.

The intervention was implemented between April and October. However, the students were on holidays in July, resulting in six months of intervention. Schools that doubled the PE classes augmented from two classes to four classes per week, increasing from 1:40 h of PE classes to 3:20 h of PE classes per week for four months. The workshop with the PE teachers consisted of five lectures, lasting 4 h on average. Each workshop was structured to present pedagogical and health-related topics to the PE teachers. After the five workshops, an additional session took place to receive feedback on the teachers’ experiences regarding the implementation and development of the contents in the PE classes. Table 1 describes the subjects introduced in each of the five workshops. In summary, the research team decided on the most relevant pedagogical and health-related topics for the workshops with the PE teachers. The third intervention group (workshop + doubling PE classes) implemented the same protocol described in the two abovementioned intervention groups. Schools in the control group maintained their habitual routine regarding their PE classes, which consisted of 1:40 PE h per week. Figure 3 presents a detailed description of the timeline of the intervention and measurements.

### 2.4. Measures

Only the variables included in the present study will be detailed in this manuscript. Baseline data were collected during March and April in 2017 (summer and autumn). The postintervention measures occurred between October and December in 2017 (spring and summer). All the measures were performed by a trained group of researchers that consisted of Master’s and PhD students and PhDs from the University of Pernambuco.

### 2.5. Primary Outcome

Academic performance was assessed by two different tests in mathematics consisting of 10 questions each. The score of each question depended on their level of difficulty. The academic performance score was the standardised sum of the 10 questions, ranging from 0 to 10 points. The questions for the academic performance tests were withdrawn from the database of questions from the Ministry of Education. This database contains questions used in the national surveys evaluating the educational system in Brazil [24].

### 2.6. Covariates

We used a questionnaire to obtain information on sex (male, female), age, and maternal education level (≤8 years of education, >8 years of education). Maternal educational is a proxy measure of the students’ socioeconomic status and it was used as an adjustment in the main analysis.

### 2.7. Statistical Analyses

All statistical analyses were conducted in STATA 15 for windows (StataCorp LP, College Station, TX, USA). The Fisher chi-square test assessed differences in the proportion of boys and girls and maternal educational level (<8 years of education and 8+ years of education) between intervention groups. One-way ANOVA evaluated differences in the variances of age by the intervention group. The Bonferroni post hoc analysis was used to indicate differences between groups. 

The linear mixed model evaluated the effects of the intervention groups on the academic performance after the intervention period. The analysis was adjusted for academic performance at the baseline, sex, age, maternal educational level (≤8 years of education, >8 years of education), the structural condition of the sports court (with and without roof coverage), and the cluster structure of the data (individuals nested within classrooms). Subgroup analyses evaluated the effect of the intervention on the participants’ academic performance at postintervention by the age group (in years) using the linear mixed model. The subgroup analysis was adjusted for academic performance at the baseline, sex, maternal educational level (≤8 years of education, >8 years of education), the structural condition of the sports court (with and without roof coverage), and the cluster structure of the data (individuals nested within classrooms). We accepted a 5% error in all analyses. 

## 3. Results

From the 1200 students included in the study, 56.42% were females. Most of the students were between 14 and 16 years of age (89.6%), whereas 3.6% were 13 years of age, and 6.8% were older than 16 years of age at baseline. Students from the workshop with the PE teachers group were younger than their peers in the other groups at baseline (Table 2).

**Figure 4:**
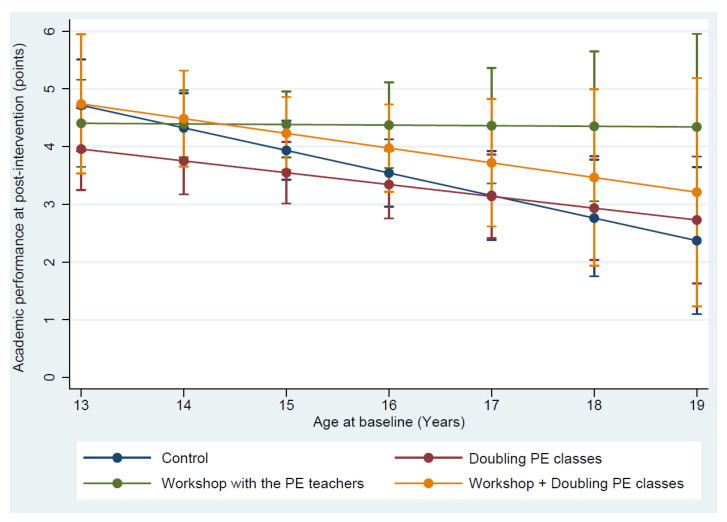
Predicted academic performance at postintervention of students from the first year of high school according to the intervention group and age. Legend: Adjusted for academic performance at the baseline, sex, maternal educational level (≤8 years of education, >8 years of education), the structural condition of the sports court (with and without roof coverage), and the cluster structure of the data (students nested within classrooms).

The academic performance at baseline was similar across groups. The intervention was not effective in increasing the academic performance of the participants (Table 3). However, subgroup analyses showed that one intervention arm increased the academic performance of the students who had failed an academic year (students older than 15 years at baseline). More specifically, we did not observe group differences in the academic performance of the students younger than 16 years of age. From 16 years of age, students from the workshop intervention group presented higher academic performance in comparison to their peers in the doubling PE classes (1.3 points on average) and the control groups (1.4 points on average). The academic performance was always similar between the workshop and the workshop + doubling PE classes intervention groups (Figure 4). See Table 4 for a detailed description of the predictive academic performance for each age and intervention group.

## 4. Discussion

Overall, none of the intervention arms, in comparison to the control group, increased the academic performance of the students. However, providing a workshop for the PE teachers positively affected the academic performance of the students who had failed an academic year in comparison to their peers in the control group. Moreover, we observed that a workshop updating pedagogical and health-related topics for PE teachers seemed a better approach in improving the academic performance than doubling the amount of PE classes for students who failed an academic year. At the end of the academic year, students who had failed an academic year (older than 15 years of age) presented lower academic performance compared to the younger peers, except for students exposed to the workshop with the PE teachers group, in which the academic performance was similar for older and younger students.

Resaland et al. (2016) [17] did not observe an effect of a multicomponent intervention on academic performance. However, authors reported that students with the poorest academic performance at baseline benefited from the effects of the intervention, which consisted of three different actions aiming at increasing the students’ physical activity level during school and at home. In addition, another study that implemented a quasi-experimental multicomponent intervention reported higher math scores in low income elementary school children [19]. It is possible that students who needed the most help are the ones benefiting from the interventions. 

Our results reinforce this theory. Students over 15 years of age in the beginning of the first year of high school were students who failed an academic year. Although, the academic performance at baseline did not differ between older and younger students, we observed that in the end of the curricular year older students had lower academic performance in comparison to the youngest peers, except for students exposed to the workshop with the PE teachers group (Figure 4). The grade retention is harmful for the social, emotional, and self esteem of the students [25]. Students who fail an academic year have a low self image and popularity with their peers. Repeaters often do not get social support and become more reserved and unfriendly with their class fellows [26]. In addition to updating the teachers in regards to several pedagogical skills, one of the workshop sessions dealt with the teachers on how to promote a greater integration of these students in the physical education classes. It is possible that students in the workshop intervention group felt more integrated and this may have helped their academic performance.

Importantly, failing an academic year has short- and long-term consequenes. There is a higher chance of dropping school and developing behavioural problems [23,27] for those students. Moreover, academic failure is associated with higher rates of infertility, mortality, and unemployment (see the 2018 World Development Report for review [23]). In addition, students with lower grades have lower earnings even nine years after finishing high school [28]. Therefore, the impact of upgrating the PE teacher skills on academic performance for students who failed an academic year should be further evaluated because of the potential attenuating effects on well-known consequences of academic failure.

Overall, our intervention did not affect the academic performance of the students independent of their age. Bugge et al. (2018) observed no improvements in academic performance in Danish students in an intervention that provided training sessions for the PE teachers in addition to exposing students to 4.5 h of PE lessons a week, whereas students in the control schools were exposed to 1.5 h of PE lessons a week [13]. Bugge et al. (2018) [13] suggested that the lack of improvements in physical activity and aerobic fitness was one of the main reasons for the nonsignificant effect on academic performance in their investigation.

In line with this theory, previous observational and controlled laboratory interventional studies observed a relationship between physical activity, aerobic fitness, and academic performance [2,4,7,8,29]. More specifically, Coe et al. (2006) only observed higher academic performance in children who met the recommendation for vigorous physical activity in comparison to children who did not perform any vigorous physical activity [15]. It is possible that effects on academic performance are dependent on improvements in physical activity and aerobic fitness levels.

Most multicomponent interventions suffered in improving students’ academic performance [13,16,17,18,19], although studies that trained teachers to incorporate physical activity in their lessons (i.e., mathematics or language) showed encouraging effects on academic performance [30,31]. School-based interventions that both increased the time in the PE classes and enhanced PE classes by either training PE teachers or by specialised professionals showed conflicting results [10,12,13,14,16]. Our results suggested that single intervention strategies seemed more effective in improving the students’ academic performance.

### Strengths and Limitations

Some of the highlighted strengths in our study include the randomised design, the large sample size, and the national standardised tests as a measure of the students’ academic performance. However, some limitations should be considered in the interpretation of our results. First, the study evaluated a short intervention period. Ideally, a longer intervention could have enhanced the interventions effects and other differences could have been observed between the intervention groups. Second, we did not assess the follow-up intervention effects in the current study. Third, we did not assess the content of the physical education lessons during the intervention period. Finally, adding other academic subjects as measures of academic performance would have been ideal. 

## 5. Conclusions

Overall, the intervention was not effective in improving the students’ academic performance. However, the workshop with the PE teachers improved the academic performance of students who had failed an academic year. It seems that providing a workshop for the PE teachers is an effective approach in avoiding the lower academic performance of the older students at the end of the curricular year. Based on our results and the existing literature, we believe that enhancing the pedagogical skills of the teachers is a promising path for improving the students’ academic performance, especially the ones who need it the most.

## Figures and Tables

**Table 1:** Date and content of the workshop sessions for the workshop with the PE teachers and the workshop + doubling PE classes intervention groups.

Date of the Sessions	Topics	
	Pedagogical	Health-Related
2 June, 2017	Current scenario and future prospects of the physical education in Brazil	Physical activity and physical fitness in the physical education classes
30 June, 2017	Challenges in the physical education classes in high school	Sedentary behaviours
28 July, 2017	Designing objectives and selecting topics (educational contents)	The teenage brain
1 September, 2017	The importance of methodological strategies and process evaluation	Mental health and psychosocial stress indicators
22 September, 2017	The importance of the motivational atmosphere and the perceived competence for the development and learning processes	Health eating and sleep quality
27 October, 2017	Session for receiving feedback on the teachers’ experiences regarding the implementation and development of the contents in the PE classes	

**Table 2:** Descriptive characteristics of 1200 students in the first year of high school at baseline according to the intervention group. Values are numbers and (percentages) unless stated otherwise.

Title	Doubling PE Classes	Workshop with the PE Teachers	Workshop + Doubling PE Classes	Control	Total	*p*
Age, in years Mean (SD)	15.08 (1.19)	14.66 (0.97)	15.07 (0.76)	15.15 (1.02)	14.99 (1.04)	<0.001 *
Female	201 (51.80)	177 (57.84)	133 (57.83)	166 (60.14)	677 (56.42)	0.149 ^§^
<8 years of maternal education	139 (41.49)	93 (34.07)	84 (39.62)	108 (43.55)	424 (39.70)	0.130 ^§^

Legend: * One-way ANOVA test; ^§^ Fisher chi-square test.

**Table 3:** Academic performance score in relation to the intervention group at baseline and postintervention and the adjusted difference of the academic performance at postintervention.

Intervention Group	Academic Performance at Baseline, Mean (SD)	Academic Performance at Postintervention, Mean (SD)	Adjusted Difference in Academic Performance at Postintervention in Relation to the Control Group*			ICC
			Coefficient	(95% CI)	*p*	
Control (*n* = 188)	3.50 (1.81)	3.64 (2.26)				0.092
Doubling PE classes (*n* = 242)	3.65 (1.87)	3.82 (2.16)	−0.355	(−1.121 to 0.410)	0.363	
Workshop with the PE teachers (*n* = 198)	3.89 (2.08)	4.45 (2.70)	0.361	(−0.387 to 1.110)	0.345	
Workshop + doubling PE classes (*n* = 152)	3.62 (1.93)	3.80 (2.01)	0.292	(−0.496 to 1.079)	0.468	

Legend: * Adjusted difference in scores of the academic performance at postintervention between the intervention groups and the control group; adjusted for academic performance at the baseline, sex, age, maternal educational level (≤8 years of education, >8 years of education), the structural condition of the sports court (with and without roof coverage), and the cluster structure of the data (students nested within classrooms).

**Table 4:** Predicted academic performance of the first year high school students at postintervention (points), standard deviations (SD), and 95% confidence intervals (CI) for each age and intervention groups.

Age	Intervention Group	Predicted Academic Performance	(95% CI)	Difference in Relation to the Control
13	Control	4.72	(3.92 to 5.51)	
	Doubling PE classes	3.96	(3.25 to 4.67)	−0.76
	Workshop with the PE teachers	4.40	(3.65 to 5.16)	−0.31
	Workshop + doubling PE classes	4.74	(3.53 to 5.95)	0.02
14	Control	4.33	(3.73 to 4.92)	
	Doubling PE classes	3.75	(3.17 to 4.33)	−0.57
	Workshop with the PE teachers	4.39	(3.82 to 4.97)	0.07
	Workshop + doubling PE classes	4.48	(3.65 to 5.32)	0.16
15	Control	3.93	(3.42 to 4.45)	
	Doubling PE classes	3.55	(3.01 to 4.08)	−0.39
	Workshop with the PE teachers	4.38	(3.81 to 4.96)	0.45
	Workshop + doubling PE classes	4.23	(3.60 to 4.86)	0.30
16	Control	3.54	(2.96 to 4.13)	
	Doubling PE classes	3.34	(2.76 to 3.93)	−0.20
	Workshop with the PE teachers *	4.37	(3.63 to 5.12)	0.83
	Workshop + doubling PE classes	3.98	(3.22 to 4.73)	0.43
17	Control	3.15	(2.38 to 3.92)	
	Doubling PE classes	3.14	(2.42 to 3.86)	−0.01
	Workshop with the PE teachers *	4.36	(3.36 to 5.36)	1.21
	Workshop + doubling PE classes	3.72	(2.62 to 4.82)	0.57
18	Control	2.76	(1.75 to 3.77)	
	Doubling PE classes	2.93	(2.04 to 3.83)	0.17
	Workshop with the PE teachers *	4.35	(3.05 to 5.65)	1.59
	Workshop + doubling PE classes	3.47	(1.94 to 4.99)	0.70
19	Control	2.37	(1.10 to 3.65)	
	Doubling PE classes	2.73	(1.63 to 3.83)	0.36
	Workshop with the PE teachers *	4.34	(2.73 to 5.95)	1.97
	Workshop + doubling PE classes	3.21	(1.23 to 5.19)	0.84

Legend: * Higher academic performance in comparison to the doubling PE classes and control groups.

## Data Availability

The dataset related to this manuscript can be made available under a reasonable request.

## Abbreviations

PE refers to physical education.
